# Prevalence of gastrointestinal stromal tumour (GIST) in the United Kingdom at different therapeutic lines: an epidemiologic model

**DOI:** 10.1186/1471-2407-14-364

**Published:** 2014-05-24

**Authors:** Justyna M Starczewska Amelio, Javier Cid Ruzafa, Kamal Desai, Spiros Tzivelekis, Dominic Muston, Javaria Mona Khalid, Philip Ashman, Andrew Maguire

**Affiliations:** 1 Health Economics and Epidemiology, Evidera, Metro Building, 6th floor, No.1 Butterwick, London W6 8DL, UK; 2 Global Market Access, Bayer Pharma AG, Berlin S157, 03, 305, Germany; 3 Health Economics, Bayer plc, Strawberry Hill, Newbury RG14 1JA, UK

**Keywords:** Epidemiology, Prevalence, Gastrointestinal stromal tumour, GIST, Model, Ultra-orphan disease

## Abstract

**Background:**

The prevalence of patients with gastrointestinal stromal tumourgst (GIST) who fail currently available treatments imatinib and sunitinib (third-line treatment-eligible GIST) is unknown, but is expected to be below an ultra-orphan disease threshold of 2/100,000 population used in England and Wales. Our study was designed to estimate the prevalence and absolute number of UK patients with unresectable/metastatic GIST at first-, second- and eventually third-line treatment.

**Methods:**

Our open population model estimates the probability that the prevalence of UK third-line treatment-eligible GIST patients will remain under the ultra-orphan disease threshold. Model parameters for incidence, proportion of unresectable/metastatic disease and survival estimates for GIST patients were obtained from a targeted literature review and a UK cancer register. The robustness of the results was checked through differing scenarios taking extreme values of the input parameters.

**Results:**

The base-case scenario estimated a prevalence of third-line treatment-eligible GIST of 1/100,000 and a prevalence count of 598 with a 99.9% likelihood of being below the ultra-orphan disease threshold. The extreme scenarios, one-way and probabilistic sensitivity analyses and threshold analysis confirmed the robustness of these results.

**Conclusions:**

The prevalence of third-line treatment-eligible GIST is very low and highly likely below the ultra-orphan disease threshold.

## Background

Gastrointestinal stromal tumours (GIST) are relatively rare soft tissue mesenchymal tumours occurring in the gastrointestinal tract, originating in the interstitial cells of Cajal involved in the regulation of the digestive system [1,2]. Recent estimates of GIST annual incidence quoted in the United Kingdom (UK) range from 1.32–1.50 per 100,000 population, although none appear population-based [3,4], equivalent to approximately 800–900 new cases each year [4]. There have been no UK prevalence studies of GIST published to date, although reports from western Sweden and Hong Kong have estimated prevalence at 12.9 per 100,000 [5] and 13.4–15.6 per 100,000 [6], respectively. Epidemiologic estimates of GIST in the UK have improved since the diagnostic coding of GIST was introduced in the third edition of the International Classification of Disease for Oncology (ICD-O), in 2000. Studies conducted before this period used the terms leiomyoma, leiomyosarcoma or leiomyoblastoma to identify GIST [2], likely resulting in overestimates. Some GIST have been diagnosed as benign and malignant mesenchymal lesions, tumours of the autonomic and peripheral nervous systems, or benign and malignant tumours not further classified; hence, these terms have been used sometimes to identify patients with a potential GIST diagnosis [5].

Currently only resection, dependent on early stage diagnosis, offers a potential cure. The proportion of patients who are not eligible for resection and have metastatic/unresectable disease at diagnosis has been reported in the United States (US), Canada and other European populations as varying between 13% and 50% [4,7-15]. The National Institute for Health and Care Excellence (NICE) imatinib appraisal has described the proportion of patients with metastatic/unresectable GIST as between 10% and 30% [4]. For these patients, two lines of systemic therapy were introduced: first-line imatinib and second-line sunitinib in 2001 and 2006, respectively. Among patients who experience both imatinib and sunitinib treatment failure (referred to here as ‘third-line treatment-eligible GIST’), current treatment options are limited to further imatinib rechallenge at increased dose, participation in clinical trials or best supportive care [16].

The prevalence of GIST patients eligible for third-line treatment in the UK is not known, but is expected to be low and potentially below orphan and ultra-orphan disease thresholds. In the European Union (EU), an orphan disease is defined as having a prevalence of less than 50 per 100,000 [17]; in England and Wales, NICE has suggested that conditions with a prevalence of less than 2 per 100,000 population may be considered ultra-orphan [18]. Given the importance of GIST prevalence on orphan or ultra-orphan disease status, our study was designed to estimate the prevalence and absolute number (i.e., prevalence count) of UK patients with unresectable/metastatic GIST at first-, second- and eventually third-line treatment. We developed an open-population model that estimated the number of subjects at each stage of treatment based on model parameters for GIST incidence, proportion of unresectable/metastatic GIST and survival estimates for GIST patients. Data to inform the model parameters were based on targeted literature review and data from a UK cancer register.

## Methods

### Data sources

To calculate the incidence of GIST, data were requested from all 11 cancer registers in the UK. Cancer registers responded to our query but only one register made data available: the West Midlands Cancer Intelligence Unit (WMCIU) from 2007 to 2010, owing to the recent introduction and variability in uptake of the specific ICD-O diagnostic code for GIST across UK cancer registers [19]. The WMCIU makes anonymized and aggregated data publically accessible to researchers for a fee to cover the expenses associated to data extraction, and acknowledgement of its contribution. The WMCIU age and gender strata incidence rates were applied to the UK population age and gender distribution to obtain a 2010 UK standardised incidence rate. The total estimated UK population in 2010 is 62,262,100 [20]. WMCIU GIST incidence estimates were consistent with available published literature, therefore supporting the quality of the data obtained from the registry [3-5,8,11,19,21].

Other model parameters were obtained from a targeted literature review. These included: the proportion of metastatic or unresectable GIST; post-resection GIST relapse rates; progression-free survival (PFS) or time to tumour progression (TTP) on first-line treatment (imatinib); PFS or TTP on second-line treatment (sunitinib) and overall survival (OS) post-imatinib and -sunitinib treatment failures. For each of these parameters several references were screened from the literature. Internal validity of the specific parameter estimates obtained from the literature, or the degree to which the cited studies are free from systematic error, is supported by the peer review process of such studies and the consistency of their results, that also supports external validity or generalizability to the UK. External validity is also expected since the documented parameter value ranges were narrow and the parameters’ base-case were chosen around mid-range values.

Sources of published data used to inform the model parameters are given in Table 1. While some data sources provided values in the units required by the model, others needed transformation. For example, survival figures in a given treatment state expressed as percent of surviving patients at a specific time (month or year) after treatment initiation required transformation into yearly rates before they could be used in the model. While we focused our literature searches on publications reporting results among UK patients, we considered it adequate to include publications from studies in the US, Canada and other European populations to increase the robustness of our model. To account for background mortality we used data from the 2010 UK Life Tables available through the UK Office for National Statistics [20] weighted by the West Midlands register GIST patient’s age distribution (2007–2010) [19] and assumed that within 10-year age bands of patients, the incidence of GIST did not vary. WMCIU data and literature search strategy are described in the Additional file 1: Appendices.

**Table 1:** Model parameters

**Parameter**	**Base-case Value (SE)**^**$**^	**Min-max range for one-way SA**^**$**^	**Distribution for PSA**^**$**^	**References**
**GIST**^**$**^**incidence (Γ) in person-years**	1.053/100000 (SE = 0.139/100000)	0.52/100000 to 1.50/100000	Gamma	[3-5,8,11,19,21,31,32]
**Proportion of resectable GIST (p)**	0.8 (SE = 0.05)	0.50 – 0.90	Beta	[4,9,11-13,15,33]
**Post-resection GIST TTT**^**$**^**(γ**_**2**_)*	0.0464 (SE = 0.0025)	0.029 – 0.186	Gamma	[34-38]
**Imatinib-treated GIST TTT (γ**_**3**_)*	0.351 (SE = 0.103)	0.205 – 0.645	Gamma	[14,39-44]
**Sunitinib-treated GIST TTT (γ**_**4**_)*	0.974 (SE = 0.085)	0.533 – 1.435	Gamma	[45-48]
**Third-line treatment-eligible GIST survival (γ**_**5**_)*	0.904 (SE = 0.10)	0.439 – 1.066	Gamma	[24,25,49-53]
**Yearly background mortality**	0.0314 (SE = 0.0023)	0.0269 – 0.0359	Gamma	[20]

^$^*GIST* = gastrointestinal stromal tumour; *SE* = standard error; *SA* = sensitivity analysis; *PSA* = probabilistic sensitivity analysis; *TTT* = time to transition;

*Yearly rate assuming patients’ survival followed an exponential distribution.

### Model overview

**Figure 1:**
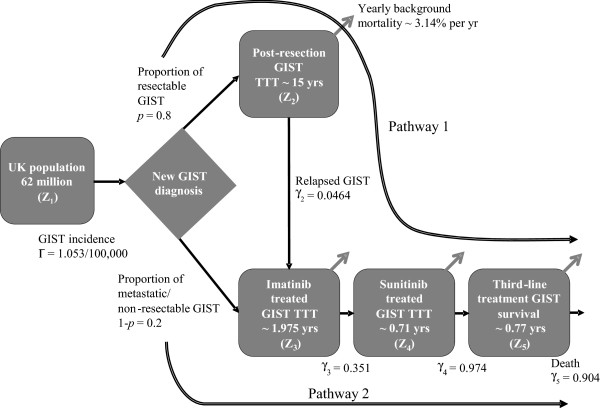
**Model to assess GIST patient prevalence in the UK at different treatment stages.** GIST = gastrointestinal stromal tumour; TTT = time to transition; UK = United Kingdom. The model parameters are given in Table 1. Z_1_ refers to the total UK population and is fixed in all calculations at 62,262,000. Z_2_ is the number of patients with non-metastatic GIST who have been resected and are currently recurrence free. Z_3_ is the number of patients with non-resectable metastatic GIST and who are currently progression free while receiving treatment with imatinib. Similarly, Z_4_ and Z_5_ are the numbers of patients who are progression-free while receiving sunitinib and a third-line treatment, respectively. γ_2_ is the annual rate of relapse which is estimated from the duration of relapse-free survival in this state. γ_3_ is the annual rate of failure calculated from the duration of PFS and TTP on imatinib. γ_4_ is the annual rate of failure calculated from the duration of PFS and TTP on sunitinib. γ_5_ is the annual probability of failure calculated from the duration of OS on a range of investigational third-line treatments or best supportive care. Γ = incidence of newly diagnosed GIST. p = proportion.

We constructed a model for calculating the prevalence of GIST in the UK population (Figure 1) that represents the flow of patients with GIST from first diagnosis to death via two possible treatment pathways and four treatment states.

In the first pathway, the newly diagnosed patients with resectable GIST have their stromal tumour removed surgically and remain in a recurrence-free state for several years before some of them relapse. At this point those who relapse enter a state of non-resectable metastatic GIST and are assumed to receive successful treatment with imatinib for approximately two years and, during this time, they are assumed to be progression-free. Following the failure of imatinib treatment, patients receive sunitinib in a second progression-free state with duration of approximately nine months. Finally, patients enter a third state during which they receive a presumed third line of treatment. They will eventually exit this final state as they will die. The model also allows patient exit through the background mortality in each of the states.

In the second pathway, patients who are metastatic or unresectable at diagnosis move directly into the imatinib treatment state joining other patients from the first treatment pathway. From this point, all patients follow the same treatment pathway until death. The flow through the model is governed by difference equations which calculate the annual number of patients presenting with GIST and numbers on each treatment type (see Additional file 1: Appendices). The model parameters correspond to rates of progression of patients through the different treatment states. Rates of progression are derived from what we called ‘time to transition’ (TTT) from one state to the next. TTT corresponds to the total period of time on the current treatment as well as any period of discontinuation upon treatment failure until the next treatment line is initiated. TTT was therefore inferred from studies reporting PFS or time to tumour progression for all states, except for third-line treatment-eligible GIST for which we used OS.

### Model parameters, analysis and sensitivity analysis

The model base-case scenario was defined by base-case values for each model parameter. All seven parameters in the model require four values to be specified (Table 1). The first is a base-case parameter estimate, while the remaining three (minimum value, maximum value, standard error) are necessary to express the uncertainty around the base-case value. Parametric uncertainty analyses were conducted through one-way and probabilistic sensitivity analysis (PSA) for the base-case and alternative scenarios. Univariate or one-way sensitivity analysis consisted of varying each parameter individually between its assumed minimum and maximum values, which were informed by the literature review, while fixing all other parameters at their base-case estimate. Thus, parameters which are most influential on model results can be identified.

The PSA consisted of varying all parameter values simultaneously by sampling each parameter 5,000 times from statistical distributions, which are specified by the mean (base-case parameter estimate) and standard error of each parameter. The beta distribution is valued between 0 and 1 and is employed in the model to represent the proportion of subjects at diagnosis with resectable GIST. The gamma distribution is strictly positively valued and is suited to model event rates, representing the rates of transition between each of the treatment states. In Table 1 we specified the preferred source for the base-case value and its standard error for each model parameter to obtain base-case estimates and to perform PSA, which provided credibility intervals for each model outcome.

Given the importance of the choice of two of the base-case values on model results, three alternative scenarios were defined based on higher GIST incidence, higher survival assumptions for third-line treatment-eligible GIST and higher for both incidence and third-line treatment-eligible GIST survival. The three aforementioned alternative scenarios were chosen by combining extreme values of the two most influential parameters from the results of the univariate sensitivity analyses.

We also performed threshold analysis. We explored what the incidence of GIST and the survival of third-line treatment-eligible GIST patients (two most influential parameters) would need to be to reach the 2 per 100,000 population prevalence figure corresponding with NICE ultra-orphan disease threshold. Threshold analysis was performed to address this question for each of the two parameters, while fixing all other parameter values.

### Model outcomes

Model outcomes for each scenario included absolute number and prevalence per 100,000 population of all patients with GIST, patients in first-line (receiving imatinib) and second-line (receiving sunitinib) treatment and third-line treatment-eligible GIST patients who experienced imatinib and sunitinib treatment failures. Each outcome was expressed with 95% credibility intervals whose upper and lower limits were determined by the 2.5% and 97.5% percentiles of the outcomes obtained from the PSA. The PSA also permitted estimation of the probability that overall and third-line treatment-eligible GIST populations met orphan or ultra-orphan disease thresholds used in the UK.

## Results

### Scenario analysis

The base-case and alternative scenarios 1, 2 and 3 are presented in Table 2. Under the assumptions of the model, the base-case scenario indicates that the prevalence of third-line treatment-eligible GIST is estimated as 1.0 per 100,000 population (95% confidence interval [CI]; 0.7–1.3), and the probability that this figure will remain below 2.0 per 100,000 population is 99.9%. In addition, the prevalence of the total GIST population is estimated as 15.0 per 100,000 population (95% CI; 11.2–19.8). The total number of patients living after suffering GIST (all stages) is estimated at 9,365 (95% CI; 6,953–12,325) and with third-line treatment-eligible GIST is 598 (95% CI: 432–804). Our model estimated the absolute number of patients receiving imatinib as 1,422 (95% CI; 838–2,368) and receiving sunitinib as 599 (95% CI: 435–789).

**Table 2:** Scenario results produced by the proposed model, with 95% confidence intervals (CI)

	Third-line treatment-eligible GIST* population prevalence per 100000 persons (95% CI)	**Probability that third-line treatment-eligible GIST population is below 2 per 100000 persons**	**Absolute number third-line treatment-eligible GIST population (95% CI)**	**Total GIST population prevalence per 100000 persons (95% CI)**	**Absolute number total GIST population (95% CI)**	**Absolute number of GIST patients on imatinib (95% CI)**	**Absolute number of GIST patients on sunitinib (95% CI)**
**Base-case Scenario GIST Incidence: 1.053/100000 person-years (p-y) Third-line treatment-eligible GIST survival: 0.77 yrs**	0.96 (0.69 – 1.29)	99.9%	598 (432 – 804)	15.04 (11.2 – 19.8)	9365 (6953 – 12325)	1422 (838 – 2368)	599 (435 – 789)
**Alternative Scenario 1 GIST Incidence: 1.5/100000 p-y Third-line treatment-eligible GIST survival: 0.77 yrs**	1.37 (1.06 – 1.73)	99.6%	851 (662 – 1080)	21.9 (13.9 – 31.7)	13364 (10697 – 16383)	2020 (1,252 – 3,258)	855 (670 – 1067)
**Alternative Scenario 2 GIST Incidence: 1.053/100000 p-y Third-line treatment-eligible GIST survival: 1.5 yrs**	1.53 (1.00 – 2.29)	90.6%	954 (622 – 1428)	15.6 (11.6 – 20.3)	9699 (7227 – 12633)	1410 (832 – 2338)	602 (437 – 802)
**Alternative Scenario 3 GIST Incidence: 1.5/100000 p-y Third-line treatment-eligible GIST survival 1.5 yrs**	2.18 (1.50 – 3.18)	37.9%	1357 (933 – 1984)	22.3 (17.7 – 27.4)	13886 (1068 – 17069)	2020 (1259 – 3282)	854 (674 – 1061)

*GIST = gastrointestinal stromal tumour.

By comparison, the first alternative scenario, which assumes a higher yearly incidence of GIST of 1.5 per 100,000 population [4], provides a prevalence estimate of third-line treatment-eligible GIST eligible patients of 1.4 per 100,000 population (95% CI; 1.1–1.7) with a corresponding probability of remaining below the 2.0 per 100,000 population threshold of 99.6%. Figures for the second alternative scenario, which assumes a longer third-line treatment-eligible GIST survival of 1.5 years, are higher than those of the base-case and the first alternative scenario with respect to third-line treatment-eligible GIST prevalence and lower in terms of probability of the third-line treatment-eligible GIST prevalence remaining below 2.0 per 100,000 population (threshold). The third alternative scenario, which assumes both higher incidence and longer third-line treatment-eligible GIST survival, produces a third-line treatment-eligible GIST prevalence of 2.2 per 100,000 population (95% CI: 1.5–3.2) and a probability of 37.9% that the third-line treatment-eligible GIST prevalence will remain below the threshold.

### Univariate sensitivity analysis

**Figure 2:**
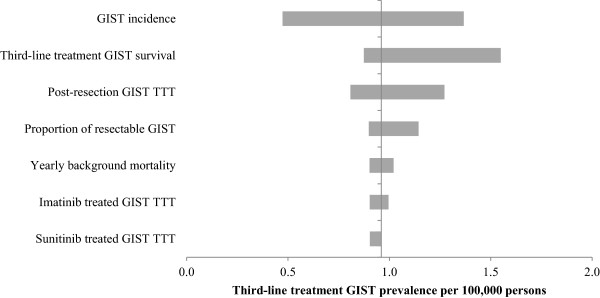
**Tornado diagram: Univariate sensitivity analysis for base-case scenario (low incidence and short third-line treatment-eligible GIST survival).** GIST = gastrointestinal stromal tumour; TTT = time to transition.

Univariate sensitivity analysis performed on the base-case and alternative scenarios demonstrate that the initial incidence of GIST and third-line treatment-eligible GIST survival were most influential on the prevalence of third-line treatment-eligible GIST, as illustrated in the tornado diagram (Figure 2). Model parameters associated with survival on imatinib and sunitinib had the least influence on prevalence of third-line treatment-eligible GIST.

### Threshold analysis

To better quantify the risk associated with the two most influential parameters, we determined the threshold incidence and third-line treatment-eligible GIST survival that would cause the third-line treatment-eligible GIST prevalence to exceed 2.0 per 100,000, while all other parameters were fixed at the base-case values. This analysis showed that the incidence of GIST would need to be 2.2 per 100,000 population or higher for the third-line treatment-eligible GIST population to exceed the threshold. The third-line treatment-eligible GIST transition rate would have to be 0.31 or higher, corresponding to a median survival after imatinib and sunitinib failure of at least 2.2 years.

## Discussion

We employed a modelling approach to estimate the overall number and prevalence of metastatic/unresectable GIST patients in the UK and those who failed on currently licensed treatments. The model demonstrated that, under a plausible set of assumptions (base-case scenario) of disease incidence and survival, the prevalence of third-line treatment-eligible GIST is 1.0 (95% CI; 0.7–1.3) per 100,000, with a 99.9% likelihood of being below the 2 per 100,000 population threshold for ultra-orphan disease status in the UK. As data values reported in published studies which informed our model parameters are subject to great variation, we examined three alternative sets of assumptions.

When we raised the assumed yearly incidence of GIST to the highest levels reported in Western countries (1.5 per 100,000 persons), the probability that third-line treatment-eligible GIST prevalence remained below the ultra-orphan disease threshold was virtually unchanged (99.6%). This scenario may be plausible given that estimates of the annual incidence of GIST may rise with improved diagnosis of tumours that are tested for c-KIT.[22] Because a new effective treatment line would prolong life in the third-line treatment-eligible GIST and therefore increase prevalence, it was important to assess the longer patient survival impact on the third-line treatment-eligible GIST prevalence. By assuming 1.5 years of survival in the third-line treatment-eligible GIST state, we estimated a 90.6% probability that its prevalence was below the ultra-orphan disease threshold. Only when assuming both higher GIST incidence and longer third-line treatment-eligible GIST survival we obtained a 37.9% probability of remaining below the threshold of interest.

Sensitivity analysis showed that initial GIST incidence and third-line treatment-eligible GIST survival were the most influential variables on the third-line treatment-eligible GIST prevalence estimate, while threshold analysis showed that only under extreme assumptions of these two parameters (GIST incidence greater than 2.2 per 100,000 population or third-line treatment-eligible GIST survival greater than 2.2 years) would the number of third-line treatment-eligible GIST patients surpass the UK ultra-orphan disease threshold. We did not explore further other model parameters in this respect. Varying estimates of post-resection GIST TTT, imatinib-treated GIST TTT, sunitinib-treated GIST TTT and the proportion of resectable GIST did not greatly influence our study findings.

The model predicted the total number of patients living after suffering GIST in the base-case to be 9,365 (95% CI; 6,953–12,325) and the number of GIST patients taking imatinib as 1,422 (95% CI; 838–2,368) or sunitinib as 599 (95% CI: 435–789).

The understanding of the aetiology and management of GIST has evolved since it was first differentiated among other soft tissue sarcomas and two lines of therapy became available for patients with metastatic or unresectable tumours. However, for a proportion of patients, these therapies eventually fail and patients who exhaust their treatments are left with re-challenging with higher imatinib dose or best supportive care. Several potential third-line treatment drugs [23-25] could be candidates for orphan or ultra-orphan disease treatment status. Orphan and ultra-orphan disease status have implications on how public funding supports the provision of treatments, and the speed of access to new treatments for suitable patients. There could be a case for potential treatments after imatinib and sunitinib failure given the low number of patients at third-line treatment-eligible GIST.

The major limitation of the study is the face validity of the model structure and the structure’s inherent assumptions on treatment pathways; however, this study presents a model, understood as a simplified and imperfect description of reality, which estimates the number of subjects at each stage of GIST treatment, based on model parameters available from the literature. Another limitation we encountered was that data needed to inform the model parameters were sparse or unsuitable, which has also been reported in economic modelling studies [26-30]. This limitation is common in modelling studies since it is not always possible to inform all of the model parameters, considering that the samples of the available studies are small [27], the evidence for the model is obtained from only one study [26] or data are used from other countries and applied to the country of interest due to lack of local evidence [29].

Some of the reviewed studies reported on important prognostic variables (i.e., mitotic count, genetic markers) that identify heterogeneous subgroups within the GIST patient population. Our model did not explicitly account for these subgroups as this would have necessitated stratum-specific TTT estimates that were mostly unavailable. As a result, we have given preference to publications with large sample sizes where a pool of GIST patients with a mix of those variables can occur, that is, studies with heterogeneous patient populations (i.e., different treatment histories, stages of GIST) and response criteria. Nonetheless, sensitivity and scenario analysis showed that the importance of data uncertainty mattered mainly for initial incidence of GIST and third-line treatment-eligible GIST survival.

We acknowledge that the model’s structure, where all patients transition through the treatment states sequentially, and assuming that patients cannot skip a particular treatment line, can be questioned. Another modelling study included up to seven plausible treatment pathways for patients with GIST which also depended on limited data for the proportions of patients following each pathway, and required assumptions for death rates and state transition probabilities [4]. We also assumed GIST as not curable, so all subjects diagnosed with GIST could not exit the GIST population. Nevertheless, the post-resection GIST TTT are widely distributed and a long post-resection GIST TTT (longer than the life expectancy) can be assumed as cured GIST. Both of these assumptions are conservative since patients remain in the model for longer, therefore, leading to overestimation of GIST-related state prevalences. Our model did not explicitly include pathways for adjuvant and neoadjuvant use of tyrosine kinase inhibitors. These therapeutic strategies can be considered a combination of the two proposed model pathways.

## Conclusion

Despite the study limitations, there is a very high probability of third-line treatment-eligible GIST prevalence being below 2.0 per 100,000 population in the UK, therefore, remaining below the ultra-orphan disease threshold. This is relevant because provision of orphan and ultra-orphan disease status can affect the speed of access to new treatments for suitable patients.

## Abbreviations

CI: Confidence interval; EU: European Union; GIST: Gastrointestinal stromal tumours; ICD-O: International Classification of Disease for Oncology; NICE: National Institute for Health and Care Excellence; OS: Overall survival; PFS: Progression-free survival; PSA: Probabilistic sensitivity analysis; TTP: Time to tumour progression; TTT: Time to transition; UK: United Kingdom; US: United States; WMCIU: West Midlands Cancer Intelligence Unit.

## Competing interests

JMSA, JCR, KD, JMK and AM were employees of Evidera (JMSA, JMK and AM are currently former Evidera employees). Evidera is a global scientific and medical affairs organization that partners with life science companies and professionals.

ST, DM and PA were Bayer employees. (ST, PA and JCR are currently former Bayer employees). Bayer is a global enterprise with core competencies in the fields of health care, agriculture and high-tech materials. Bayer is conducting research on GIST therapy.

Bayer sponsored this research.

## Authors’ contributions

AM conceived the study; JMSA coordinated the study; JMSA conducted the literature search and extraction; JCR and KD participated in the literature search and extraction; JMK collected data from registers; JMSA, JCR and KD developed the model structure; AM, ST, DM and PA reviewed the model structure; KD carried out the model analyses (parameter estimation, sensitivity analyses and threshold analyses); JCR carried out additional data analyses; AM and JMK participated in additional data analyses; JMSA, JCR and KD drafted different sections of the manuscript; AM, JMK, ST, DM and PA helped to develop the draft manuscript; all authors reviewed and provided feedback to the manuscript, and all authors read and approved the final manuscript.

## Authors’ information

JMSA (PhD) Observational Research Manager at Amgen

JCR (MD, DrPH, MBA, MSc) Research Scientist and Epidemiologist, Epidemiology and Database Analytics at Evidera

JMK (PhD) Associate Director, Epidemiology at Takeda Pharmaceuticals

AM (MSc, FSS) Director, Epidemiology at OXON Epidemiology

KD (PhD) Research Scientist, Health Economics at Evidera

ST (MSc) Director, Global Value Access & Policy at Amgen

DM (MSc) Senior Health Economist at Bayer plc

PA (PhD) Senior Vice President and Managing Director at Alimera Sciences.

## Pre-publication history

The pre-publication history for this paper can be accessed here:

http://www.biomedcentral.com/1471-2407/14/364/prepub

## Supplementary Material

Additional file 1: Appendices **Appendix I.** Method Used to Calculate Incidence of GIST in UK. **Appendix II.** Model Specifications. **Appendix III.** Key Data Sources for Model Parameters. **Appendix IV.** Literature Search Strategy [54-58]. Click here for file
