# Supplementary material for: Prevalence of gastrointestinal stromal tumour (GIST) in the United Kingdom at different therapeutic lines: an epidemiologic model
Source: BMC Cancer. 2014 May 24;14:364. doi: 10.1186/1471-2407-14-364 (PMC4039646; doi:10.1186/1471-2407-14-364)
Supplement: Additional file 1: Appendices — Appendix I. Method Used to Calculate Incidence of GIST in UK. Appendix II. Model Specifications. Appendix III. Key Data Sources for Model Parameters. Appendix IV. Literature Search Strategy [54-58]. [file 1471-2407-14-364-S1.docx]

**Additional file 1**

**Appendix I – Method Used to Calculate Incidence of GIST in UK**

*Annual Incidence Rates*

Cases of GISTs diagnosed in the West Midlands from 2007 to 2010 were extracted from the cancer dataset by sex and age band.

Gender- and age-specific rates for groups of interest were calculated:

$$Gender and age specific rate i =\frac{No of GIST in i gender and age group}{Population of i gender and age group in West Midlands}\left( 1,000,000 \right)$$

Gender- and age-specific rates were standardised to the UK population using the direct method for standardisation.

The gender- and age-specific rates were then projected to the population of UK:

$$Projected No of cases for i gender and age group=\frac{gender and age specific rate for i\mathrm{group}}{1,000,000}\left( the population of i gender and age group in UK \right)$$

$$\mathrm{Estimated} \mathrm{standardised} \mathrm{incidence} \mathrm{rate} \mathrm{of} \mathrm{GIST} \mathrm{in}\mathrm{UK}=\frac{sum of projected cases across all groups}{2010 population in UK}\left( 1,000,000 \right)$$

The UK age- and gender-standardised incidence rate estimated from the register is 10.53 per 1,000,000 person-years. This includes both invasive cancers and cancers of uncertain behaviour. Confidence intervals for this rate were computed using standard methods [[54](#_ENREF_54)].

**Appendix II – Model Specifications**

The model is illustrated in Figure 1 in the main body of this report. The model parameters are given in Table 1. Z_1_ refers to the total UK population and is fixed in all calculations at 62,262,000. Z_2_ is the number of patients with non-metastatic GIST who have been resected and are currently recurrence free. Z_3_ is the number of patients with non-resectable metastatic GIST and who are currently progression free while receiving treatment with imatinib. Similarly, Z_4_ and Z_5_ are the numbers of patients who are progression-free while receiving sunitinib and a third-line treatment, respectively.

The number of patients who are disease free at year *t*+1 following resection is given by:

$$Z_{2}^{t+1}=Z_{2}^{t}+p\Gamma Z_{1}^{t}-\gamma_{2}Z_{2}^{t}-\delta Z_{2}^{t}$$

The first term $Z_{2}^{t}$ refers to the number of subjects remaining in the current state from the previous year. The second term *p*${\Gamma Z}_{1}^{t}$ is the number of newly diagnosed cases of GIST who are added to this state, where *Γ* refers to the incidence of newly diagnosed GIST and *p* is the proportion of those who are resectable. The third term γ_2_$Z_{2}^{t}$ is the number of patients who relapse with metastatic GIST thus exiting the relapse-free state, where γ_2_ is the annual rate of relapse which is estimated from the duration of relapse-free survival in this state (explained in detail in parameter section). The final term δ$Z_{2}^{t}$ is the number of patients who exit the current state due to background mortality, where δ is the background death rate (also explained in the parameter section).

The number of subjects who are in a progression-free state while receiving imatinib in year *t*+1 is given by:

$$Z_{3}^{t+1}=Z_{3}^{t}+(1-p)\Gamma Z_{1}^{t}+\gamma_{2}Z_{2}^{t}-\gamma_{3}Z_{3}^{t}-{\delta Z}_{3}^{t}$$

The first term $Z_{3}^{t}$ refers to the number of subjects remaining in the current state from the previous year. The second term (1−*p*)*Γ*$Z_{1}^{t}$ is the number of newly diagnosed cases of unresectable or metastatic GIST who are added this state, where *Γ* refers to the incidence of newly diagnosed GIST and 1−*p* is the proportion of those who are unresectable or metastatic. The third term γ_2_$Z_{2}^{t}$ is the number of previously disease-free resectable patients who begin treatment with imatinib following GIST relapse after surgery. The fourth term γ_3_$Z_{3}^{t}$ is the number of patients who depart the progression-free state due to failure of imatinib, where γ_3_ is the annual rate of failure calculated from the duration of PFS and TTP on imatinib (explained in the parameter section). The final term δ$Z_{3}^{t}$ is the number of patients who exit the current state due to background mortality.

The number of subjects who are in a progression-free state while receiving sunitinib in year *t*+1 is given by:

$$Z_{4}^{t+1}=Z_{4}^{t}+\gamma_{3}Z_{3}^{t}-\gamma_{4}Z_{4}^{t}-{\delta Z}_{4}^{t}$$

The first term $Z_{4}^{t}$ refers to the number of subjects remaining in the current state from the previous year. The second term γ_3_$Z_{3}^{t}$ is the number of patients who depart the progression-free state due to failure of imatinib and begin treatment on sunitinib. The third term γ_4_$Z_{4}^{t}$ is the number of patients who depart the progression-free state due to failure of sunitinib, where γ_4_ is the annual rate of failure calculated from the duration of PFS and TTP on sunitinib (also explained in the parameter section). The final term δ$Z_{4}^{t}$ is the number of patients who exit the current state due to background mortality.

The number of subjects who are in a progression-free state while receiving third-line treatment in year *t*+1 is given by:

$$Z_{5}^{t+1}=Z_{5}^{t}+\gamma_{4}Z_{4}^{t}-\gamma_{5}Z_{5}^{t}-{\delta Z}_{5}^{t}$$

The terms in this equation are explained in a manner similar to the previous equations. The third term $\gamma_{5}Z_{5}^{t}$ is the number of patients who depart the progression-free state due to failure of the presumed third-line of treatment, where γ_5_ is the annual probability of failure calculated from the duration of OS on a range of investigational third- line treatments or best supportive care (see the parameter section). As a consequence, patients exit the current state due to GIST-related mortality. The final term${\delta Z}_{5}^{t}$is the number of patients who exit the current state due to background mortality (mortality from all other causes except GIST).

**Appendix III – Key Data Sources for Model Parameters**

| **Parameter** | **Source** | **Comment** |
| --- | --- | --- |
| Annual incidence  of GIST | [[19](#_ENREF_19), [55](#_ENREF_55)] | WMCIU is a population-based cancer register covering 5.3 million people in England, or one tenth of the UK population, with a variety of social and ethnic backgrounds, representative of the whole UK [[55](#_ENREF_55)]. The WMCIU currently operates a registry that receives data from 28 acute hospitals, 17 private hospitals, 9 hospices, some community hospitals and general practitioners. Approximately 40,000 new cases of cancer are notified per year totalling an excess of 1.2 million records. The WMCIU’s registration and data quality teams ensure information is entered into the database in a timely and accurate manner. The population distributions of the areas covered by the WMCIU and England have similar age distributions and results extrapolated from the WMCIU may be generalizable to the population of England. Data from the WMCIU have been extensively published in scientific journals.  All available data from years 2007–2010 using GIST-specific ICD-O codes were requested in March 2012 from WMCIU (n=230). The ICD morphology code for GISTs appeared in version 3 of ICD-O, and migration from version 2 has been slow and varied across the regional registries. Currently, of the eleven UK registries only one registry, the West Midlands Cancer Intelligence Unit (WMCIU), uses the specific GIST code and has only started to use the specific code in recent years (since 2000, with complete recording from 2007). Age and sex standardisation was performed to extrapolate figures to the entire UK population, to obtain an annual incidence rate of 1.053 per 100,000 persons (standard deviation [SD] 0.139 per 100,000). Thus, 95% of values sampled from a Gamma distribution for incidence are expected to range between 0.781–1.325 per 100,000 in the PSA (SD 0.139 per 100,000). |
|  | Ahmed et al., 2008 [[3](#_ENREF_3)] | Estimated 1.32/100,000 persons based on case identification (n=225) from two Nottingham hospitals between years 1987 and 2003. |
|  | Hislop et al., 2010 [[4](#_ENREF_4)] | Provided an incidence of 1.5/100,000 which was quoted in a report from Scotland [[56](#_ENREF_56)] that in turn cited a reference from an American Society of Clinical Oncology (ASCO) 2003 oral presentation [[57](#_ENREF_57)] possibly originated from Sweden. NOTE: For Scenario 2, the base-case is 1.5/100,000, but no CIs were given by Hislop et al. [[4](#_ENREF_4)]. Arbitrarily, this was chosen to be 0.225. |
|  | Brabec et al., 2009 [[8](#_ENREF_8)];  Mucciarini et al., 2007 [[11](#_ENREF_11)];  Goettsch et al., 2005 [[21](#_ENREF_21)];  Nilsson et al., 2005 [[5](#_ENREF_5)]; Tryggvason G et al., 2005 [[32](#_ENREF_32)]; Rubió J et al, 2007 [[31](#_ENREF_31)] | European rates varied between 0.52/100,000 person-years in Czech Republic and Slovakia (n=278) [[8](#_ENREF_8)], 0.7 in Italy (n=124) [[11](#_ENREF_11)], 1.1 in Iceland (n=57) [[32](#_ENREF_32)] and Spain (n=46) [[31](#_ENREF_31)], 1.3 in Netherlands in 2003 (n=206) [[21](#_ENREF_21)] and 1.45 in Sweden (n=288) [[5](#_ENREF_5)]. Since published studies on UK and European incidence indicated values ranging between 0.52–1.50 per 100,000 these specified the minimum and maximum values for the one-way sensitivity analysis. |
| GIST Resectability | Witkowski et al., 2011 [[33](#_ENREF_33)] | Reported US nationwide trends from the SEER database 1998–2007 using GIST-specific code to identify 3,604 patients. For the 2002–2007 period the proportion of patients recommended for surgery decreased from 85.5% to 80.1%. |
|  | Pisters et al., 2011 [[13](#_ENREF_13)] | Reported data from 122 sites from 2004 through 2009 with 882 patients included in the register, an observational database to understand the management of patients with GIST in the USA. Among 719 patients with localised GIST at diagnosis, the most common first-line treatment was surgery for 87%. The initial treatment for 50.3% patients with metastatic disease was surgery. |
|  | Perez et al., 2006 [[12](#_ENREF_12)]; | Perez et al. reports on 1,696 patients from two registries in the USA with 900 among them (53%) being localized. |
|  | Hislop et al., 2010 [[4](#_ENREF_4)] | Quoted a range of figures of GIST unresectability among UK GIST patients, 10% to 30% |
|  | Aparicio et al., 2004 [[7](#_ENREF_7)];  Brabec et al., 2009 [[8](#_ENREF_8)];  Mucciarini et al., 2007 [[11](#_ENREF_11)];  Braconi et al., 2008 [[9](#_ENREF_9)];  Rutkowski et al., 2007 [[14](#_ENREF_14)]  Bumming et al. 2006 [[10](#_ENREF_10)] | European populations gave estimates of unresectability: Aparicio et al. [[7](#_ENREF_7)] noted 42.7% of incomplete resections or metastatic disease among French GIST patients at a single institution (n=59); Brabec et al. [[8](#_ENREF_8)] reported 30.9% metastatic GISTs in the Czech and Slovak GIST register; Mucciarini et al. [[11](#_ENREF_11)] reported that 21.8% of GIST patients in the Modena Cancer Register, Italy had unresectable/metastatic disease; Braconi et al. [[9](#_ENREF_9)] looked at patients at a single Italian institution (n=104) and reported 15% of metastatic tumours at first presentation; Rutkowski et al. [[14](#_ENREF_14)] reported 24% of metastatic GISTs in a Polish Clinical GIST Register (n=335); Bumming et al. [[10](#_ENREF_10)] reported on GIST patients in western Sweden (n=259) and noted that there were 85.3% of complete resections meaning that 14.7% GIST patients were either metastatic or unresectable. |
| Post-resection GIST TTT | Joensuu et al., 2012 [[34](#_ENREF_34)] | Base-case value chosen from Joensuu et al. [[34](#_ENREF_34)] included data from 10 series of patients totalling 2,560 patients. Recurrence-free survival (RFS) after surgery showed 62.9% (n=235) at 10 years (95% CI 59.8–66.0). This corresponds to a median survival of 15.0 years (95% CI 13.48–16.68) and an annual recurrence rate of 0.0464 per year (95% CI 0.0415–0.0514). These median survival figures were transformed to rates, assuming patients survival followed an exponential distribution according to: S(t) = e ^– λ t^ . Applying this equation to the median survival of 15 years gives 0.5 = e ^– λ 15^, and solving for λ gives λ = 0.0464. Choosing a base-case for annual rate of recurrence 0.0464 with an SE of 0.00248 implies that 95% of values sampled from a Gamma distribution for the annual rate are expected to range between 0.0417 and 0.0514. Other publications report ranges from 3.7 to 24 years median survival, so the min-max values for the rate = 0.0286–0.1863. |
|  | Gold et al., 2009 [[35](#_ENREF_35)];  Rutkowski et al., 2011 [[36](#_ENREF_36)];  DeMatteo et al., 2008 [[37](#_ENREF_37)];  Mochizuki et al., 2004 [[38](#_ENREF_38)]; | Gold et al. [[35](#_ENREF_35)] reported on a group of 127 Memorial Sloan-Kettering Cancer Center GIST patients used to construct a nomogram and two groups of GIST patients (Mayo Clinic series and GEIS series). There were 42 patients among 127 who had recurrence with a median follow-up of patients free from recurrence of 4.7 years. The RFS ranged from 63% to 78% at 5 years in the three datasets used in this study.  Rutkowski et al. [[36](#_ENREF_36)] reported on a series of 640 consecutive patients from a prospectively collected tumour register with median disease-free survival after resection of 57 months and the estimated 5-year relapse-free survival rate of 50% (95%CI: 45–56%).  DeMatteo et al. [[37](#_ENREF_37)] report data that allows estimates of median RFS of 3.7 to 7.5 years.  A total of 127 patients with localised primary GIST who underwent complete gross surgical resection were studied from 1983 to 2002.  With a median follow-up for patients free of recurrence of 4.7 years, median RFS was not reached with 83% at 1 year, 75% at 2 years, 63% at 5 years, and 60% at 10 years.  Mochizuki et al. [[38](#_ENREF_38)] report data (n=60) that can result in median RFS of 24 years. The median time to the detection of recurrence was 20 months (range 5–80 months), and 5 of the 8 recurrences (62.5%) occurred within 2 years of surgery. |
| Imatinib-treated GIST TTT | Azribi et al., 2009 [[39](#_ENREF_39)] | Base-case value chosen from Azribi et al. (n=36) [[39](#_ENREF_39)] included 23.7 months of median PFS (12.9–34.4).  23.7 months corresponds to an annual progression rate  of 0.351 (CI: 0.2418–0.6448). Choosing a base-case for annual rate of recurrence 0.351 with an SE of 0.101 implies that 95% of values sampled from a gamma distribution for the annual rate are expected to range between 0.181 and 0.575. |
|  | Demetri et al., 2002 [[40](#_ENREF_40)]; Blanke et al., 2008 [[41](#_ENREF_41)];  Bonvalot et al. 2006 [[42](#_ENREF_42)];  Rutkowski et al., 2007 [[14](#_ENREF_14)];  Al-Batran et al., 2007 [[43](#_ENREF_43)];  Cohen et al., 2009 [[44](#_ENREF_44)]; | Other studies supported the chosen model values.  Demetri et al. [[40](#_ENREF_40)] and Blanke et al. [[41](#_ENREF_41)] reported on a randomised clinical trial with 147 patients who received imatinib (400 mg or 600 mg). Median time to progression (TTP) was 24 months overall (95% CI: 17–30). This corresponds to an annual progression rate of 0.347 (95% CI: 0.277–0.488).  Bonvalot et al. [[42](#_ENREF_42)] reported a median PFS of 18.7 months among 180 French patients who underwent surgery following imatinib treatment and PFS was 23.4 months among patients with planned tumour resection.  Another study in 335 Polish patients with advanced inoperable/metastatic GIST treated with imatinib 400–800 mg daily was reported by Rutkowski et al. [[14](#_ENREF_14)]. They reported a median PFS 40.5 months.  Al-Batran et al. [[43](#_ENREF_43)] reported a median PFS of 18.9 months (1–43.5+ months) among 38 German patients with metastatic GIST receiving imatinib therapy.  Similar results have been reported in two open-label, controlled, multicentre, randomised Phase III studies (n=946 and n=746). Median PFS time was approximately 20 months [[44](#_ENREF_44)]. |
| Sunitinib-treated GIST TTT | Reichardt et al., 2008 [[45](#_ENREF_45)];  Blay et al., 2009 [[47](#_ENREF_47)] | Base-case value for TTT in patients on sunitinib was chosen from Reichardt et al. [[45](#_ENREF_45)] who reported a median TTP of 37 weeks (95% CI: 35–44) from 1,091 patients, which correspond to an annual TTP rate of 0.974 (CI 0.819–1.029). Choosing a base-case annual rate of TTP of 0.974 and SE=0.085 implies that 95% of values sampled from a gamma distribution for the annual TTP rate are expected to range between 0.814 and 1.147.  The same study has been reported by Blay et al. [[47](#_ENREF_47)] and an updated median survival of 41 weeks was provided from 1,117 patients. |
|  | Demetri et al., 2006 [[46](#_ENREF_46)];  Raut et al., 2010 [[48](#_ENREF_48)] | Demetri et al. [[46](#_ENREF_46)], in a pivotal placebo controlled study of 312 patients, reported a median TTP for the intention to treat population as 27.3 weeks (0.53 years) and median PFS as 24.1 weeks (0.46 years) for the sunitinib arm.  A study by Raut et al. [[48](#_ENREF_48)] among 50 USA GIST patients on sunitinib undergoing surgery for metastatic GIST reported median PFS after surgery was 5.8 months (0.48 years) and after start of sunitinib 15.6 months (1.30 years). |
| Third-line treatment GIST survival | Italiano et al., 2012 [[49](#_ENREF_49)] | Base-case value for end-stage TTT was chosen from Italiano et al. (n=223) [[49](#_ENREF_49)]. Median OS of 9.2 months (CI 7.5–10.9) corresponds to an end-stage annual mortality rate of 0.904 (CI: 0.76–1.11). Choosing a base-case annual mortality rate mortality of 0.904 and SE=0.1 implies that 95% of values sampled from a gamma distribution are expected to range between 0.719 and 1.11.  NOTE: For Scenario 3 the end-stage value is 1.5 years corresponding to annual rate of 0.462. SE=0.15675. |
|  | Reichardt et al., 2010 [[50](#_ENREF_50)];  Nishida et al., 2009 [[58](#_ENREF_58)];  Montemurro et al., 2009 [[52](#_ENREF_52)];  Trent et al., 2011 [[25](#_ENREF_25)];  Kindler et al., 2011 [[24](#_ENREF_24)];  Wiebe et al., 2008 [[53](#_ENREF_53)] | Several other studies have reported OS of investigational third-line treatments in patients who are resistant or intolerant to imatinib and sunitinib, ranging from 0.65 to 1.58 years [[24](#_ENREF_24), [25](#_ENREF_25), [50-52](#_ENREF_50)], with one study reporting median OS on best supportive care of 0.78 years (choice to continue or stop imatinib or sunitinib) [[50](#_ENREF_50)].  Reichardt et al. [[50](#_ENREF_50)] noted a significant difference in median OS was observed: 405 vs. 280 days between the study arm treated with the new investigational drug (nilotinib) (n=132) and treated with best supportive care (n=65), respectively.  Two other studies by Nishida et al. (n=35) [[58](#_ENREF_58)] and Montemurro et al. (n=52) [[52](#_ENREF_52)] in patients also treated with nilotinib reported median OS of 72 weeks (310 days) and 34 weeks (95% CI 3–65; range 2–135), respectively.  Another investigational drug (dasatinib) has been studied in a Phase II trial by Trent et al. (n=50) [[25](#_ENREF_25)] and median OS was reported as 19 months.  Kindler et al. (n=38) [[24](#_ENREF_24)] and Wiebe et al. (n=26) [[53](#_ENREF_53)] reported a median OS in patients treated with sorafenib of 11.6 months (95% CI: 8.8–14.3) and 13.0 months (95% CI: 5.1– ∞), respectively. |

GIST: Gastrointestinal stromal tumour ICD-O: International Classification of Diseases for Oncology

CI: Confidence interval SE: Standard error

PSA: Probabilistic sensitivity analysis SD: Standard deviation

OS: Overall survival PFS: Progression-free survival

RFS: Recurrence-free survival WMCIU: West Midlands Cancer Intelligence Unit

TTT: Time to transition TTP: Time to progression

SEER: Surveillance epidemiology and end results ASCO: American Society of Clinical Oncology

GEIS: Grupo Español de Investigación en Sarcomas or Sarcoma Research Spanish Group

**Appendix IV – Literature Search Strategy**

We conducted a targeted search for available literature in English language that would provide supporting information to our model. In our searches we included epidemiological studies, clinical trials, health technology assessments and other studies of relevance. We excluded case reports, editorials, letters, and comments. We also searched for grey literature and information on the governmental institution’s websites.

The searches screened were compiled based on four components which generated the pool of abstracts to be reviewed for their relevance.

1. Gastrointestinal stromal tumour (American and British English): **'gastrointestinal stromal tumour'**, **'gastrointestinal stromal tumour'**, **'gastrointestinal stromal tumour'**, **'gastrointestinal autonomic tumour'**, **'gastrointestinal autonomic tumour'**
2. Epidemiology of cancer (inc.: incidence, prevalence, survival) **'cancer epidemiology'**, **'cancer epidemiology'**, **'cancer epidemiology'**, **'incidence'**, **'incidence'**, **'incidence'**, **'survival'**, **'survival'**, **'survival'**, **'prevalence'**, **'prevalence'**, **'prevalence'**
3. Use failure rates of sunitinib and imatinib **'imatinib'**, **'sunitinib'**, **'rates'**, **'use'**, **'fail'**, **'failure'**
4. Metastasis and/or unresectability: **'metastatic'**, **'metastasis'**, **'unresectable'**

The literature searches were conducted in two phases.

*Phase 1*

Phase 1 was conducted on 10 April 2011. The search was done for articles published between 2000 and 2011. This search yielded 15,357 abstracts. After abstracts were screened for relevance and duplicates removed 1,140 publications were identified as potentially relevant. During the next level screening and information extraction, 10 additional citations (referenced in the relevant publications) were identified and added to an overall pool of 129 relevant publications**.**

*Phase 2*

Phase 2 was conducted on 7 February 2012 to supplement the previous searches with any newly published material between 2011 and 2012. After abstracts were screened for relevance and duplicates removed 250 articles were deemed potentially relevant. Further screening revealed that 25 articles were relevant. In addition, we searched for conference abstracts and other relevant information and 30 additional sources were added.

During the literature review process, the following inclusion and exclusion criteria were applied:

***Inclusion Criteria***

*Disease specification:*

Gastrointestinal Stromal Tumour (GIST)

i. *Study focus:*

a. GIST epidemiology (incidence, prevalence and survival rates)

b. Rates of unresectable/metastatic disease in GIST (search for prognosis and outcomes related to GIST)

c. Rates of use of imatinib & sunitinib and the failure rates

ii. *Restrictions by countries:*

No country restriction has been applied. However, shall the sufficient number of publications be found the main focus will be on the data from the United Kingdom.

iii. *Languages:*

All reviewed articles are published in English language.

iv. *Time period*:

Due to the specificity of the topic and first ICD-O classification in year 2000 the search was limited to papers published between 2000 and 2011.

***Exclusion Criteria***

Studies were excluded if they were:

1. Not consistent with the inclusion criteria.
2. Not relevant for the issues under inquiry, e.g., when the abstract shows no apparent contents of interest.
3. Animal- or in-vitro studies.

iv. Case reports, abstracts without available full texts that do not provide sufficient information to extract data*, or enable a full assessment of study quality, letters, commentaries or editorials.

The number of references screened, retrieved and extracted, as well as the consistency of the parameter values they provided, support that the parameter used to inform the model do not provide systematic error to the study results.
